# The Diagnostic Value of High-Resolution Computed Tomography Features Combined with Mycoplasma Pneumoniae Ribonucleic Acid Load Detection for Refractory Mycoplasma Pneumonia

**DOI:** 10.1155/2022/6460865

**Published:** 2022-05-04

**Authors:** Hongping Wei, Chunyan Wang, Lili Ding, Min Wu

**Affiliations:** ^1^Department of Pediatrics, Yiwu Central Hospital, Yiwu 322000, Zhejiang, China; ^2^Department of Pharmacy, Yiwu Central Hospital, Yiwu 322000, Zhejiang, China

## Abstract

The aim of this study was to investigate the value of high-resolution computed tomography (CT) images and mycoplasma *pneumoniae* (MP) ribonucleic acid (RNA) load detection in the early diagnosis of refractory mycoplasma *pneumoniae* (RMP) and provide more methods for the diagnosis and treatment of RMP. Seventy children with MP were divided into the RMP group (H1 group, 31 cases) and the MP group (H2 group, 39 cases) according to pathological findings, and all of them underwent CT scanning. MP-RNA load and genotype distribution were analyzed in both groups, and the diagnostic efficacy of CT combined with MP-RNA load for RMP was calculated. The sensitivity of children in the H1 group to erythromycin (59.17% vs 71.56%) and clarithromycin (53.21% vs 67.03%) was lower than that in the H2 group, and the resistance rate of children in the H1 group to erythromycin (71.43% vs 67.53%) and clarithromycin (64.24% vs 50.37%) was higher than that in the H2 group (*P* < 0.05); the regression coefficients between lactate dehydrogenase (LDH) and the MPLI value of RMP were −0.064 and −0.413, respectively, which were significantly negatively correlated (*P* < 0.05); the accuracy (96.5%), sensitivity (92.5%), and specificity (88%) of CT + MP-RNA in the diagnosis of RMP were significantly higher than those of CT alone (91%, 88%, and 82%) and MP-RNA alone (88%, 84.5%, and 74%), which were significantly different (*P* < 0.05). The results of high MP-RNA load detection can be used as an indicator to predict RMP, and the diagnostic efficacy is significantly improved after combination with high-resolution CT, with high clinical application value.

## 1. Introduction

Mycoplasma *pneumoniae* (MP) is mainly transmitted through the respiratory tract, mostly in late summer and early autumn [1–3]. Refractory mycoplasma pneumonia (RMP) is usually because the body's immunity is weakened and resistance is weakened, resulting in easy recurrent infection of the patient's lungs and symptoms such as obvious fever, irritable dry cough, and dyspnea [4, 5]. Macrolides, such as roxithromycin and erythromycin, are mostly used for the clinical treatment of RMP; if it is associated with bacterial infections, levofloxacin should be taken at the same time for combination therapy; if it is accompanied by a significant decrease in saturation, systemic oxygen therapy should be given [6, 7].

The clinical manifestations of RMP lack specificity, so pathogenic testing is required for diagnosis. Among them, the separation and cultivation of MP have always been the gold standard for true MP, but unfortunately there are some disadvantages such as time-consuming and harsh operating conditions [8–10]. Although the fast culture drug susceptibility (FCDS) method can diagnose the RMP within 24 hours, it is prone to bleeding and misdiagnosis. MP antibody detection is one of the commonly used clinical detection methods, but because the antibody itself needs to be detected about a week after infection and the waiting time is too long, it is not suitable for early diagnosis [11]. In addition, another method is to use real-time fluorescent PCR to detect MR-RNA from throat swabs. Its specific high sensitivity and specificity will not be affected by objective conditions such as disease course, degree of infection, and body immunity, so it is applicable for the diagnosis of early stage of MP [12, 13]. The RMP is largely reflected in MR resistance, and the resistance mechanism at the gene level has always been a hot topic for scholars. For example, literature indicated that the resistance of mycoplasma *pneumoniae* to macrolide antibiotics is mainly due to changes in the nucleotide sequence of the ribosomal 50S subunit 23S rRNA, domain V and II regions [14]. In addition, studies have shown that bacteria can produce inactivating enzymes against macrolide antibiotics, destroy the structure of macrolide antibiotics, and cause them to lose their antibacterial activity. Therefore, it is necessary to explore the effects of MP genotype and MP-RNA load on the early stage of MP.

With the development of imaging technology, the clinical diagnosis of MP has increasingly relied on imaging methods. Computed tomography (CT) uses precisely collimated X-ray beams, gamma rays, and ultrasound together with a very sensitive detector to scan a certain part of the human body one by one. The scanning time is fast, and the image is clear, which can be used for the inspection of a variety of diseases [15]. The thickness of ordinary CT is usually 5–10 mm, which is relatively thick, so it can only be reconstructed with low spatial frequency algorithms. The layer thickness of high-resolution CT scans can reach 1–1.5 mm, and on this basis, high-resolution algorithm reconstruction can be carried out, which shows the lesions more clearly, especially some fine structures. For example, it can display the lobules and bronchi, lobular spacing, lobular arteries, and veins of the lungs. It can be further examined and diagnosed on the basis of conventional CT. In particular, when some patients find small lung nodules, they usually choose to do high-resolution CT for further diagnosis [16].

In summary, the selection of the early diagnosis plan for RMP has always been a hot issue in clinical research. Therefore, 70 children with MP were selected as the research objects. The children were subjected to mycoplasma *pneumoniae* drug sensitivity test, high-resolution CT, MP-RNA load determination, and 23S rRNA V region 2063 genotype sequencing, to explore the application value of high-resolution CT images and MP-RNA load detection in the early diagnosis of RMP.

## 2. Materials and Methods

### 2.1. Research Objects and Grouping

In this study, 70 children with MP who were admitted to the hospital from October 2018 to October 2020 were selected as the research objects. There were 43 boys and 27 girls, aged 1–8 years, with an average age of 5.21 + 1.33 years. According to the results of pathological diagnosis, the samples were divided into the RMP (H1 group, 31 cases) and MP (H2 group, 39 cases) groups. MP-RNA load and genotype distribution were analyzed in both groups. In addition, the two groups of children were suitable for CT examination and then combined with MP-RNA load detection for the diagnosis of the two groups of children. The sensitivity, specificity, and accuracy of CT images combined with MP-RNA load in the diagnosis of MP were calculated and were compared with the single diagnostic results of the two examination methods. The study had been approved by the ethics committee of hospital, and the children and their families had understood the situation of the study and signed the informed consent forms.

Inclusion criteria are as follows: children with throat swab MP-RNA copy number >500; children who received relevant drug treatment before admission; and children who had understood the experiment and signed informed consents.

Exclusion criteria are as follows: patients with common respiratory tract infections; patients with mental illness; patients who had not been reviewed in hospital after treatment; patients who withdrew from the study due to personal reasons; and patients with atypical pathogens such as *Chlamydia pneumoniae* and *Chlamydia trachomatis*.

### 2.2. Culture and Drug Sensitivity Test of Mycoplasma Pneumoniae

The specific steps were given as follows. The sterile cotton swab treated with normal saline was tossed three times on the throat of the child, and the obtained sample was immediately placed in the freezing medium and restored to a liquid state at room temperature; 50 *μ*L of sample was pipetted and dropped into the C-well of the drug-sensitive plate. The cotton swab was placed in the culture flask, stirred many times, and then squeezed against the bottle wall after taking it out; after the obtained liquid was mixed well, 50 *μ*L was pipetted and dropped into the *C* + well and other drug-sensitive holes. The drug-sensitive plate was placed in a 37 °C incubator for 24 hours. The wells of the drug-sensitive plate contained 12 kinds of antibiotics (erythromycin, azithromycin, clarithromycin, doxycycline, clindamycin, telithromycin, levofloxacin, enoxacin, sparfloxacin, grepafloxacin, gatifloxacin, and trovafloxacin). The drug resistance to microorganisms was analyzed based on the inhibition effect on growth of the microorganisms of different concentrations of antibiotics.

The interpretation criteria [17] were as follows. It can be evaluated based on the degree of discoloration of the drug-sensitive plate: it could be determined as positive result if the *C* + well of the drug-sensitive plate changed from red to light blue, and it could be determined as negative result if the color remained unchanged. If the two drug-sensitive plate wells were all red, it meant that it was sensitive to the antibiotic; one red and one yellow well meant that it was mediator; and two yellow wells meant that it was resistant to the antibiotic.

### 2.3. Fluorescence Quantitative PCR Detection

The specific steps were as follows. The sterile cotton swab treated with saline was tossed over the throat of the child three times, and then, it was squeezed to dry. 1 mL of the swab liquid was sucked into a 2 mL centrifuge tube and centrifuged at 12,000 rpm for 10 minutes to obtain the precipitate. 50 *μ*L of negative and positive quality control materials was placed into two 2 mL centrifuge tubes, which were added with 50 *μ*L of nucleic acid extract, respectively. The mixture was mixed and lysed at 110 °C for 8 minutes and centrifuged at 12000 rpm for 10 minutes to collect the supernatant (20 *μ*L) for PCR. The number of tubes required for the PCR was set as *L*, and then, *L*=Sample number+negative control +Positive control+Positive standard substance could be obtained. There were 1 negative control tube, 1 positive control tube, and 4 positive standard tubes. The *L* test tubes were added with the treated samples, MP-negative quality control substance, MP-positive quality control substance, and MP-positive quantitative standard substance (with 2 *μ*L for each). Then, the tubes were capped and centrifuged at 2000 rpm for 15 seconds. After that, the test tubes were placed into the reaction tank of the PCR instrument one by one to perform the PCR amplification according to the set cycle conditions. PCR amplification conditions were set as follows: running at 95 °C for 3 minutes, denaturation at 95 °C for 50 seconds, and annealing at 55 °C for 1 minute (above operations were repeated for 10 cycles); and running at 93 °C for 30 seconds and annealing at 55 °C for 50 seconds (the operations were repeated for 30 times).

MP-RNA load can be determined according to the following procedures. The MPLI of positive samples was calculated by taking the content of H actin-RNA as the internal reference, so MPLI=−lg(Copy quantity(*MP* − R*NA*)/Copy quantity(Hactin − R*NA*)) could be obtained. The smaller the MPLI value, the greater the MP-RNA load.

### 2.4. Sequencing

PCR was performed with reference to the primer sequence of the 23S rRNA V region in the previous literature [18, 19]. PCR amplification conditions were set as follows: denaturation at 95 °C for 5 minutes, run at 94 °C for 15 seconds, run at 52 °C for 30 seconds, and run at 72 °C for 30 seconds (above operations were cycled for 30 times), extension at 72 °C for 10 minutes, and storage at 4 °C. Then, 5 *μ*L of the PCR product was taken for agarose gel electrophoresis, recovery purification, and sequencing. The sequencing result was compared with the reference sequence of the gene bank searched by the National Center for Biological Information (NCBI) to determine the 2063 genotype in the 23S rRNA V region.

### 2.5. CT Examination

In this study, the 16-slice spiral CT was adopted to scan the patient. Conventional CT was applied to scan the chest with a slice thickness of 10 mm and a slice distance of 10 mm, and some slices were selected for high-resolution CT scanning. The scan range was from the tip of the lung to the bottom of the lung. High-resolution CT scan parameters were defined as follows: collimation was 1.25 mm × 8 mm, pitch was 1.35, 0.8 seconds was required for each circle, tube voltage was 120 kV, tube current was 120 mA, and 13.5 mm was set per circle.

### 2.6. Observation Indicators

The basic data of the two groups of children were collected and recorded, including age, gender ratio, body mass index (BMI), white blood cells (WBCs), C-reactive protein (CRP), procalcitonin (PCT), lactate dehydrogenase (LDH), fever time, hospitalized time, cough time, IgA, IgG, IgM, and imaging data (CT and X-ray film). The drug resistance and sensitivity of MP of the children in two groups were recorded, including erythromycin, azithromycin, clarithromycin, doxycycline, clindamycin, telithromycin, levofloxacin, enoxacin, sparfloxacin, grepafloxacin, gatifloxacin, and trovafloxacin. The proportions of drug resistance gene mutation and the distributions of genotype mutations (*A*, *C*, *G*, and *T*) in the two groups of children were collected and recorded.

### 2.7. Statistical Analysis

The data in this study were analyzed by SPSS 19.0 version statistical software. The measurement data were expressed as mean ± standard deviation (‾*x* ± *s*), and the counting data were given in percentage (%). Pairwise comparison adopted a one-way analysis of variance. Origin 8.0 was adopted for drawing.

## 3. Results and Discussion

### 3.1. Comparison of Clinical Characteristics of Children in Two Groups

The clinical characteristics of children in the H1 and H2 groups were compared, and the results are illustrated in Table 1. The differences in age, gender ratio, BMI, WBC, CRP, fever time, hospitalized time, cough time, IgA, IgG, and IgM levels of children in the H1 and H2 groups were not statistically obvious (*P* > 0.05); the LDH, PCT, and antipyretic time of the children in the H1 group were much shorter than those of the children in the H2 group, showing statistical differences (*P* < 0.05).

### 3.2. Imaging Characteristics of Some Children

**Figure 1:**
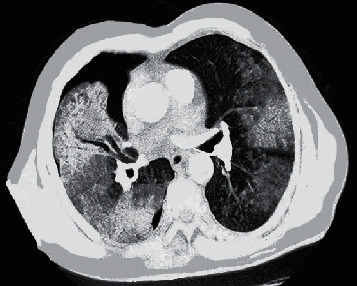
CT image of Case 1.

Figure 1 suggests the CT image of Case 1. Chest CT indicated that the lesion progressed rapidly and multiple lung-air sacs were formed.

**Figure 2:**
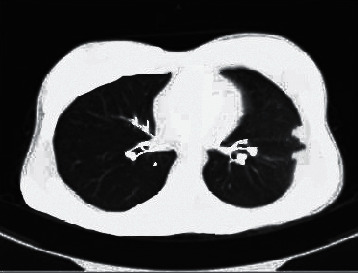
CT image of Case 2.

Figure 2 shows the CT image of Case 2. Chest CT showed acinar nodules, tree-bud sign, tree-fog sign, and lesions distributed along the bronchi, accompanied by bronchial wall thickening and peribronchitis.

### 3.3. Comparison of Drug Sensitivity Test Results of Children in the H1 and H2 Groups

**Figure 3:**
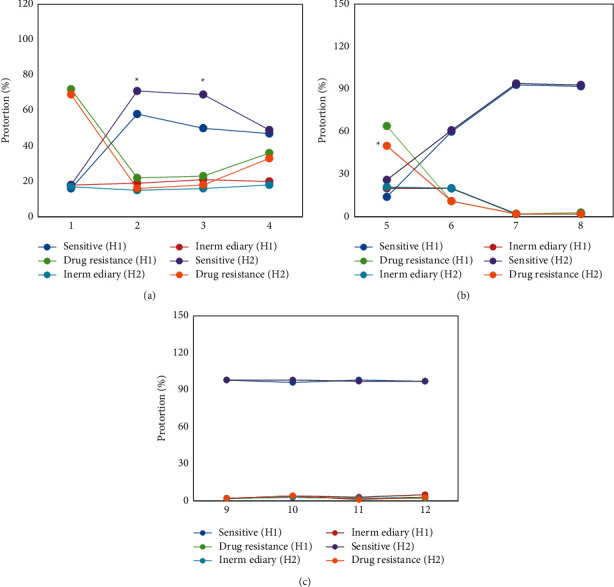
Comparison of drug sensitivity test results of children in the groups H1 and H2 (1–12 referred to erythromycin, azithromycin, clarithromycin, doxycycline, clindamycin, telithromycin, levofloxacin, enoxacin, pefloxacin, grepafloxacin, gatifloxacin, and trovafloxacin, respectively). (a) The sensitivity results of MP to erythromycin, azithromycin, clarithromycin, and doxycycline; (b) the sensitivity results of MP to clindamycin, telithromycin, levofloxacin, and enoxacin; and (c) the sensitivity results of MP to sparfloxacin, grepafloxacin, gatifloxacin, and trovafloxacin. *∗*he difference was visible statistically in contrast to the H1 group (*P* < 0.05).

Figure 3 shows the comparison of drug sensitivity test results of children in the groups H1 and H2. It illustrated that the two groups of children with MP had higher resistance rates to erythromycin (71.43% vs 67.53%) and clindamycin (64.24% vs 50.37%) and had higher sensitivity rates to azithromycin (59.17% vs 71.56%), clarithromycin (53.21% vs 67.03%), and telithromycin (62.45% vs 65.49%). Among them, children in the H1 group showed a lower sensitivity rate to azithromycin and clarithromycin than the children in the H2 group, and the differences were statistically observable (*P* < 0.05). The two groups of children with MP all had high sensitivity rates to levofloxacin, enoxacin, sparfloxacin, grepafloxacin, gatifloxacin, and trovafloxacin, all of which were greater than 90%, and the drug resistance rates were extremely low (lower than 2%).

### 3.4. Comparison of MP-RNA Load of Children in the H1 and H2 Groups

**Figure 4:**
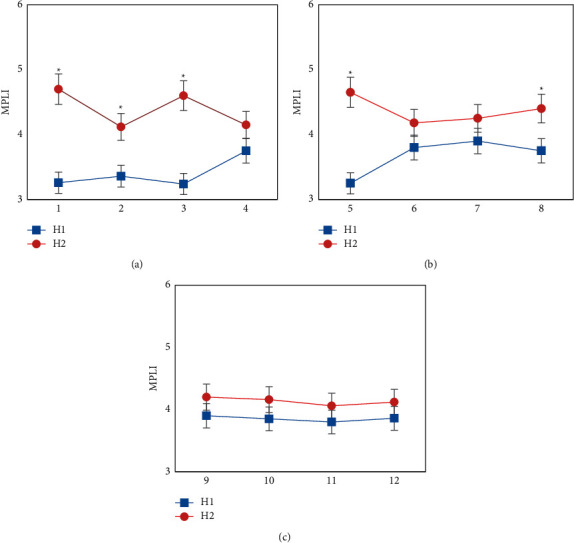
Comparison of MP-RNA load of children in the H1 and H2 groups (1–12 referred to erythromycin, azithromycin, clarithromycin, doxycycline, clindamycin, telithromycin, levofloxacin, enoxacin, sparfloxacin, grepafloxacin, gatifloxacin, and trovafloxacin, respectively). (a) showed the sensitivity results of MP to erythromycin, azithromycin, clarithromycin, and doxycycline; (b) showed the sensitivity results of MP to clindamycin, telithromycin, levofloxacin, and enoxacin; and (c) showed the sensitivity results of MP to sparfloxacin, grepafloxacin, gatifloxacin, and trovafloxacin. *∗* suggested that the difference was statistically remarkable compared with the H1 group (*P* < 0.05).

The comparison of MP-RNA load of children in the H1 and H2 groups is illustrated in Figure 4. It revealed that the MPLI values of resistance to erythromycin, azithromycin, clarithromycin, clindamycin, and enoxacin in the H1 group were greatly lower than those in the H2 group, showing statistical differences (*P* < 0.05), while the MPLI values of resistance to doxycycline, telithromycin, levofloxacin, sparfloxacin, grepafloxacin, gatifloxacin, and trovafloxacin in children in the H1 group were not greatly different from those in the H2 group, showing no statistical significance (*P* > 0.05).

### 3.5. Comparison of Gene Sequencing Results of Children in the Groups H1 and H2

**Figure 5:**
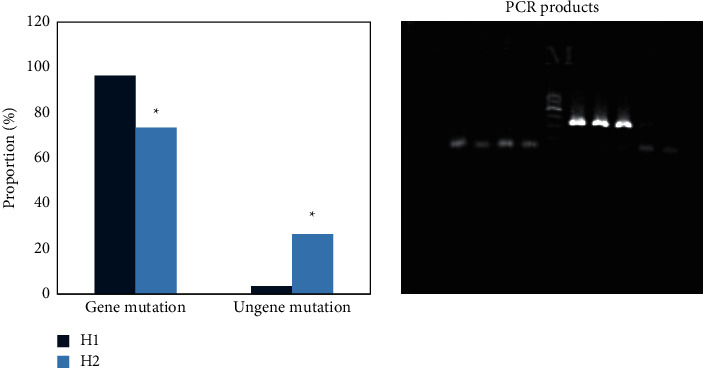
Comparison of gene sequencing results of children in the groups H1 and H2. *∗* suggested that there was a statistical difference compared with the H1 group (*P* < 0.05).

The comparison of drug resistance gene mutations in children in the H1 and H2 groups (Figure 5(a)) showed that the mutation rate of the 2063 gene in the 23S rRNA V region in the H1 group (96.42%) was greatly higher in contrast to that in the H2 group (73.51%), showing meaningful difference (*P* < 0.05); the non-gene mutation rate (3.58%) of children in the H1 group was obviously lower in contrast to the rate in the H2 group (26.49%), and the difference was remarkable (*P* > 0.05). Figure 5(b) shows the agarose gel electrophoresis of the PCR product. The rightmost was the marker from top to bottom: 600 bp, 500 bp, 400 bp, 300 bp, 200 bp, and 100 bp. On the right side of the marker was the target band. It was observed that the target band was brighter, clearly visible, and primer dimers under 200–300 bp and 100 bp.

**Figure 6:**
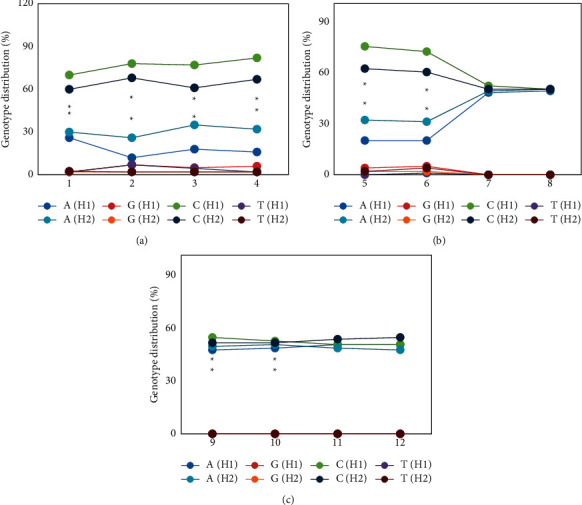
Distribution of genotype of antibiotic-resistant cases in children in the groups H1 and H2 (1–12 referred to erythromycin, azithromycin, clarithromycin, doxycycline, clindamycin, telithromycin, levofloxacin, enoxacin, sparfloxacin, grepafloxacin, gatifloxacin, and trovafloxacin, respectively). (a) showed the distribution of genotype in children with resistance to erythromycin, azithromycin, clarithromycin, and doxycycline; (b) showed the distribution of genotype in children with resistance to clindamycin, telithromycin, levofloxacin, and enoxacin; and (c) showed the distribution of genotype in children with resistance to sparfloxacin, grepafloxacin, gatifloxacin, and trovafloxacin. *∗* meant that the difference was statistically dramatic in contrast to the H1 group (*P* < 0.05).

The genotype distributions of antibiotic-resistant cases in the H1 and H2 groups are given in Figure 6. It illustrated that the erythromycin-, azithromycin-, clarithromycin-, doxycycline-, clindamycin-, and telithromycin-resistant cases showed A and *G* mutations in both groups of children, and the proportions of *G* mutation at 2063 in the H1 group of children resistant to erythromycin, azithromycin, clarithromycin, doxycycline, clindamycin, and telithromycin were obviously greater than that of the H2 group, while A mutation was the opposite, showing statistical differences (*P* < 0.05). The two groups of children showed A and *G* mutations in levofloxacin-, enoxacin-, sparfloxacin-, grepafloxacin-, gatifloxacin-, and trovafloxacin-resistant cases, but the mutation ratio was about 50%, and there was no dramatic difference between the two (*P* > 0.05).

### 3.6. Correlation of MP-RNA Load and Drug Resistance Gene Mutation to RMP

As shown in Table 2, RMP was normalized (1 referred to RMP and 0 for MP), and Pearson's correlation analysis was performed for MP-RNA load and drug resistance gene mutation. The results revealed that RMP and drug resistance gene mutation were extremely positively correlated (*r* = −0.415 and *P* < 0.001); RMP and MPLI resistance values showed an obviously negative correlation (*r* = −0.399 and *P* < 0.05).

To further explore the impacts of MPLI value and drug resistance gene mutation on RMP, different clinical indicators (LDH, PCT, and antipyretic time) and MP-RNA load and drug resistance gene mutation were incorporated into the multivariate regression analysis. As shown in Table 3, there was no obvious correlation between PCT and antipyretic time to the RMP (*P* > 0.05); the regression coefficients of RMP to LDH and MPLI were −0.064 and −0.413, respectively, and there was an extremely negative correlation (*P* < 0.05); the drug regression coefficient of resistance gene mutation to the RMP was 0.388, which showed a remarkably positive correlation (*P* < 0.05).

### 3.7. Diagnostic Performances of CT and MP-RNA Load for RMP

**Figure 7:**
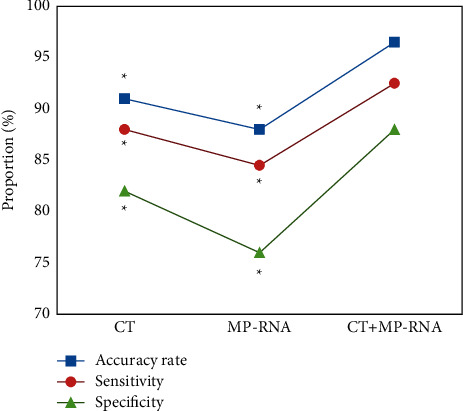
Diagnostic performances of CT and MP-RNA load for RMP. *∗* meant significant difference compared with CT + MP-RNA (*P* < 0.05).

Figure 7 shows the accuracy (96.5%), sensitivity (92.5%), and specificity (88%) of CT + MP-RNA in the diagnosis of RMP were significantly higher than those of CT alone (91%, 88%, and 82%) and MP-RNA alone (88%, 84.5%, and 74%), and the differences were significant (*P* < 0.05).

## 4. Discussion

With the widespread use of antibiotics, the drug resistance of mycoplasma *pneumoniae* has received widespread clinical attention. Since 2000, the resistance rate of mycoplasma *pneumoniae* isolates to macrolides has increased year by year, which has made the treatment of MP became difficult to be treated, based on which RMP is sourced [20, 21]. Because of the atypical early clinical manifestations of RMP, it is easy to be misdiagnosed and mistreated, so how to effectively make early diagnosis is very important. Compared with conventional CT, high-resolution CT shows clearer lesion display characteristics and a finer structure display, which can assist physicians in accurate diagnosis [22, 23]. In this study, 70 children with MP were selected as the research objects, including 31 cases of RMP (H1 group) and 39 cases of MP (H2 group). The clinical characteristics of the two groups of children were compared, and it was found that the LDH, PCT, and antipyretic time of children in the H1 group were much shorter than those of the H2 group, and the differences were statistically obvious (*P* < 0.05). Such results indicated that LDH, PCT, and antipyretic time may be related to the RMP [24]. The FCDS method found that the two groups of children's MPs were highly sensitive to azithromycin (59.17% vs 71.56%), clarithromycin (53.21% vs 67.03%), and telithromycin (62.45% vs 65.49%); the sensitivity rates of MP to azithromycin and clarithromycin in children in the H1 group were lower in contrast to the rates in children in the H2 group (*P* < 0.05). Such results were different from the findings of Mohanraj et al. [25], which may be because the abuse and irregular treatment of traditional macrolide antibiotics has reduced the sensitivity of MP to azithromycin, clarithromycin, and telithromycin, and RMP showed lower sensitivity [26]. The two groups of children with MP had higher sensitivity rates to levofloxacin, enoxacin, sparfloxacin, grepafloxacin, gatifloxacin, and trovafloxacin, all of which were higher than 90%, and the drug resistance rate was extremely low (lower than 2%), indicating that quinolone antibacterial drugs had good in vitro antimicrobial activity against mycoplasma *pneumoniae*. The results of Wei et al. [27] are consistent with the results of this exploration.

In addition, the MPLI values of children with resistance to erythromycin, azithromycin, clarithromycin, clindamycin, and enoxacin in the H1 group were lower than those in the H2 group, and the differences were great statistically (*P* < 0.05). Such results were similar to those of Lan et al. [28]. The smaller the MPLI value, the higher the MP-RNA load, which could objectively reflect the balance between MP and host immune clearance. Thus, the results indicated that RMP had a higher MP-RNA load. The proportions of *G* mutation at 2063 in children resistant to erythromycin, azithromycin, clarithromycin, doxycycline, clindamycin, and telithromycin in the H1 group were obviously greater in contrast to the proportions in the H2 group, while A mutation was the opposite, showing statistically obvious differences (*P* < 0.05). It suggested that A and *G* mutations at 2063 had greater impacts on the occurrence of RMP [29]. To further explore the impacts of MPLI value and drug resistance gene mutation on RMP, multifactor regression analysis was performed, and it was found that the regression coefficients of RMP to LDH and MPLI values were −0.064 and −0.413, respectively, showing an obviously negative correlation (*P* < 0.05); the regression coefficient of RMP to the resistance gene mutation was 0.388, showing a visibly positive correlation (*P* < 0.05). It indicated that high MP-RNA load, high drug resistance gene mutation, and low LDH levels could be deemed as indicators to predict RMP. It conforms to the conclusions of the study by Chen et al. (2021) [30]. The accuracy, sensitivity, and specificity of CT + MP-RNA in the diagnosis of RMP were significantly greater than that of single CT and MP-RNA, and the difference was statistically obvious (*P* < 0.05). This showed that high-resolution CT combined with MP-RNA load detection is better than a single detection method for the diagnosis of RMP.

## 5. Conclusion

It investigated the value of high-resolution CT images and MP-RNA load detection in the early diagnosis of RMP. The results showed that the detection results of high MP-RNA load could be used as an indicator to predict RMP, and the diagnostic efficacy was significantly improved after combining with high-resolution CT, with high clinical application value. However, there are still some shortcomings. The small sample size and single source of the selected patients resulted in a few different causes of drug resistance, which may have some impact on the results. The subsequent selection of patients will be increased for multicenter and large-sample analysis. In conclusion, the results can provide a reference for the selection of diagnostic indicators for RMP.

## Figures and Tables

**Table 1:** Comparison of clinical characteristics of children in the H1 and H2 groups.

Projects	H1 group (*n* = 31)	H2 group (*n* = 39)	t or *χ*2	P
Age (years)	5.02 ± 1.21	5.10 ± 1.03	−0.134	0.343
Male (n/%)	19/61.29	24/61.54	−0.157	0.352
Female (n/%)	12/38.71	15/38.46	−0.216	0.491
BMI (kg/m^2^)	18.67 ± 3.07	18.45 ± 3.14	−0.339	0.509
WBC (×10^9^/L)	10.28 ± 2.37	11.43 ± 2.07	0.247	0.229
CRP (mg/L)	22.09 ± 4.36	20.17 ± 5.28	0.303	0.201
PCT (ug/L)	134.89 ± 10.62	120.67 ± 11.37	5.140	0.036
LDH (U/L)	271.22 ± 50.61	225.14 ± 48.93	6.783	0.025
Fever time (d)	8.15 ± 3.62	6.89 ± 4.25	0.241	0.232
Hospitalized time (d)	12.65 ± 5.17	11.03 ± 5.09	0.212	0.267
Antipyretic time (d)	16.07 ± 4.66	11.26 ± 4.71	6.546	0.028
Cough time (d)	17.95 ± 4.78	15.64 ± 4.43	0.398	0.187
IgA (g/L)	0.98 ± 0.45	0.91 ± 0.44	−0.211	0.413
IgG (g/L)	8.89 ± 0.97	8.63 ± 0.93	0.107	0.315
IgM (g/L)	1.64 ± 0.31	1.60 ± 0.44	−0.199	0.488

**Table 2:** Correlation of MP-RNA load and drug resistance gene mutation to RMP.

Variable	RMP/MP	
	r	P
Drug resistance gene mutation	0.415	0.001
MPLI value	−0.399	0.011

**Table 3:** Multivariate regression analysis of MP-RNA load and drug resistance gene mutation to RMP.

Variable	RMP		
	Regression coefficient	*t* value	*P*
LDH	−0.064	3.461	0.027
PCT	0.139	2.552	0.059
Antipyretic time	0.266	2.815	0.071
MPLI value	−0.413	3.767	0.008
Drug resistance gene mutation	0.388	4.025	0.001

## Data Availability

The data used to support the findings of this study are available from the corresponding author upon request.
